# Magnetic Force Enhanced Sustainability and Power of Cam-Based Triboelectric Nanogenerator

**DOI:** 10.34133/2021/6426130

**Published:** 2021-03-08

**Authors:** Hakjeong Kim, Hee Jae Hwang, Nghia Dinh Huynh, Khanh Duy Pham, Kyungwho Choi, Dahoon Ahn, Dukhyun Choi

**Affiliations:** ^1^Department of Mechanical Engineering (Integrated Engineering Program), Kyung Hee University, Yongin 17104, Republic of Korea; ^2^Department of Mechanical Engineering, Korea Aerospace University, Goyang 10540, Republic of Korea; ^3^Division of Mechanical Engineering, Kongju National University, Cheonan 31080, Republic of Korea

## Abstract

Since the first invention of triboelectric nanogenerators (TENGs) in 2012, many mechanical systems have been applied to operate TENGs, but mechanical contact losses such as friction and noise are still big obstacles for improving their output performance and sustainability. Here, we report on a magnet-assembled cam-based TENG (MC-TENG), which has enhanced output power and sustainability by utilizing the non-contact repulsive force between the magnets. We investigate the theoretical and experimental dynamic behaviors of MC-TENGs according to the effects of the contact modes, contact and separation times, and contact forces (i.e., pushing and repulsive forces). We suggest an optimized arrangement of magnets for the highest output performance, in which the charging time of the capacitor was 2.59 times faster than in a mechanical cam-based TENG (C-TENG). Finally, we design and demonstrate a MC-TENG-based windmill system to effectively harvest low-speed wind energy, ~4 m/s, which produces very low torque. Thus, it is expected that our frictionless MC-TENG system will provide a sustainable solution for effectively harvesting a broadband of wasted mechanical energies.

## 1. Introduction

A “hyperconnected society” refers to the connection of all things, such as people, machines (devices), and space, wherein information is created, shared, and utilized between the connected objects [1–4]. The number of connected devices exceeded the world population in 2008, and it is expected to increase nearly four-fold by 2023 [5]. Connected devices such as PCs, TVs, smartphones, and wearable devices are already familiar, and they are connected via the Internet of Things (IoT) to provide services such as digital payments, online healthcare, and smart homes [6]. The IoT, first introduced as the US military's sensor network technology, has evolved over the past 40 years and is now commonly found in buildings, traffic systems, and smart cities [7–9]. In the IoT network, connected devices must be able to generate and transmit information anytime, anywhere.

Therefore, they must be wireless devices to have lower cost, more mobility, and more scalability than wired devices. With the development of wireless communication technology, there are various solutions for data transmission without wires [10]. To bypass another wire, the power line, most connected devices currently use batteries. However, batteries have critical limitations such as limited lifetime, the need for frequent replacement or recharging, and attendant high cost and high risk for maintenance. Therefore, a connected device needs an independent, self-powered system for improved operation time and little or no maintenance.

Triboelectric nanogenerators (TENGs) are emerging energy-harvesting devices that generate useful electric power by the movement of tribo-surfaces [11–18]. Since their debut in 2012, many novel designs and applications for harvesting energy from various mechanical sources have been introduced [19–26]. The power density has reached 1,200 Wm^−2^, the volume density has reached 490 kWm^−3^, and an energy conversion efficiency of 50%-85% has been demonstrated [27]. Hybrid nanogenerators have been also introduced to broadening the working frequency ranges by utilizing more than two energy harvesting mechanisms in one structure [28, 29]. The triboelectrification between the tribo-surfaces in a TENG is caused by their contact and separation. The movements of the tribo-surfaces can be vertical motions repeated perpendicular to the tribo-surface, or sliding motion repeated horizontally to the tribo-surface. In the latter case, a tribo-surface can be a disc or a cylindrical structure, so repetitive contact and separation are possible even with the unidirectional motion of the energy source. Furthermore, adjustment of the triboelectrification frequency can be achieved by resizing the repeated tribo-surface, such as a grating structure, resulting in higher power output. However, since a sliding contact has to be made, the selection of materials is limited due to mechanical wear, and the design and manufacture of precise motion guides are required to maintain sliding contact between the tribo-surfaces during operation. In the case of vertical motion, the structure is simple and there is no sliding, so damage between tribo-surfaces is extremely small, leading to long unit life (i.e., high sustainability). However, unlike in sliding motion, there is a disadvantage in that an energy source that repeats the movement up and down is required, and the corresponding operating frequency is relatively low because the linear contact-separation motion is normally slow. In our previous study, a mechanical cam-based TENG (C-TENG) was implemented as a contact and separation mode by using high-speed rotational energy [30]. By using a rotating cam, repetitive triboelectrification was provided for the unidirectional motion of the energy source. The advantages of long unit life and high power output were simultaneously realized by changing the number of cams, which changed the frequency of the triboelectrification.

In practical and industrial applications, the sustainability of a TENG is critical, and this is meaningful only if it has a longer lifespan than battery replacement or recharge cycles. Our C-TENG has less wear between the tribo-surfaces than a sliding-mode TENG, but it still has mechanical parts with limited life—the rotating cam and spring. Especially, the cam contact causes mechanical energy losses such as noise, impact, and further continuous wear that can lead to the destruction of the TENG (Figure S1(a-b)). More importantly, such a mechanical cam has a limitation in its pushing force: a higher pushing force produces higher friction, which can cause the cam to stick (Figure S1(c)). Herein, we propose a magnet-assembled, cam-based TENG (MC-TENG) which can provide superior sustainability and boosted output power. Instead of mechanical cam contacts, magnets provide non-contact pushing and repulsive forces to overcome the limitations of energy loss, noise, friction, and damage between the cam and the top plate. Furthermore, we can expect a significant enhancement of power output compared to a C-TENG because of the reduced contact-separation time (Δ*t*) in magnetic contact mode, and an augmented pushing force without the stuck cam problem. We investigate these behaviors with a high-speed camera and magnified the time span of the output power. Finally, all the mechanical pushing and repulsive parts of the C-TENG were replaced by permanent magnets, thus resulting in high sustainability and boosted power. We also design and demonstrate an effective MC-TENG-based windmill system to harvest energy from low-speed wind producing very low torque. Thus, our MC-TENG could be considered a cornerstone technology to improve sustainability and boost power for effectively utilizing TENGs in practical environmental conditions.

## 2. Results

### 2.1. Mechanical Behaviors of Permanent Magnets

**Figure 1:**
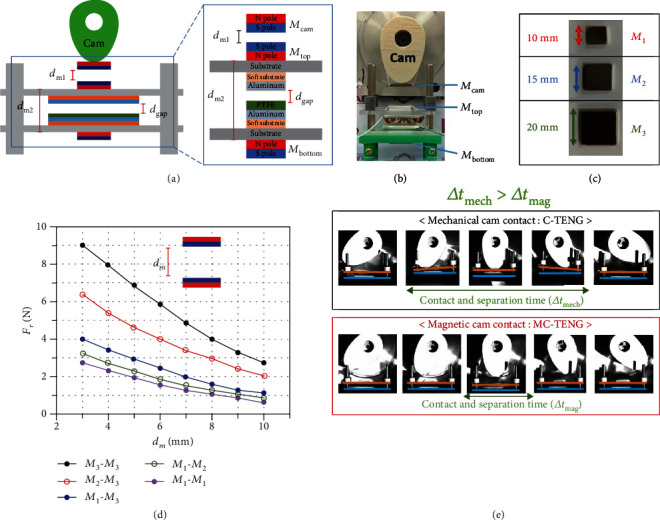
Magnet-assembled, cam-based TENG (MC-TENG). (a) Schematic illustration of an MC-TENG with permanent magnets. The inset showed a vertical-contact type TENG device including materials. (b) Photograph for MC-TENG. (c) Cuboidal magnets of different sizes. (d) Measured magnetic force for different combinations of magnets. (e) Contact-separation times (Δ*t*) for C-TENG and MC-TENG monitored by a high-speed camera.

A vertical contact-separation type of TENG generates electric power by contact and separation of the tribo-surfaces in linear direction. Since an MC-TENG utilizes permanent magnets to drive the relative motion of the tribo-surfaces (Figures 1(a) and 1(b)), the repulsive force between the magnets is an important factor. The detailed device fabrication can be found in Materials and Methods (Figure S2). As shown in Figure 1(c), three kinds of cuboidal magnets were used for experiments with the MC-TENG. They are denoted by *M*_1_, *M*_2_, and *M*_3_ according to the lengths of the square top sides, which were 10, 15, and 20 mm, respectively. The thickness of all the magnets was 3 mm. Figure 1(d) shows the measured repulsive force data of different combinations of the magnets with respect to the distance between them. As expected, when the distance decreased or the size of the magnet increased, the repulsive force increased. The detailed measurement process by the load cell can be found in Figure S3.

### 2.2. Contact-Separation Behaviors in the C-TENG and MC-TENG

As shown in Figure 1(e), when the cam is aligned to the center of the top plate, the tribo-surfaces have full contact. However, before alignment, the top plate tilts and a local contact of the tribo-surfaces occurs. Because the cam is sized to fit the distance between the motor and the top plate when the top plate is in full contact with the bottom plate, the rotating cam is forced to touch the top plate in its initial position. This local contact also occurs when the cam rotates away from the aligned position. Due to these local contacts, the contact-separation time (Δ*t*_mech_) in a C-TENG might be increased. In contrast, the magnetic force between the magnets of an MC-TENG is non-contact. The full contact of the tribo-surfaces by the non-contact magnetic force is confirmed when the cam with the magnet is aligned to the center of the top plate, as shown in Figure 1(e). In this case, the top plate does not tilt and local contact of the tribo-surfaces does not occur. Therefore, the contact-separation time (Δ*t*_mag_) in an MC-TENG is shorter than Δ*t*_mech_, which can be expected to enhance the power output from the MC-TENG.

**Figure 2:**
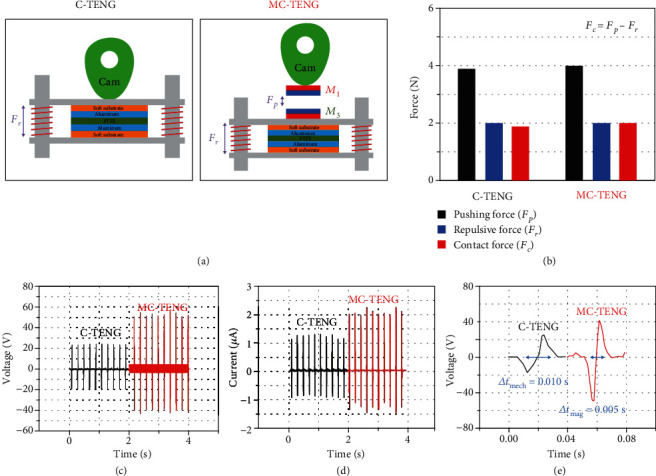
Effect of cam contact mode (i.e., mechanical and magnetic contacts). (a) Schematic illustration of a C-TENG with mechanical contact and MC-TENG with magnetic contact. (b) Comparison of forces calculated by a pushing force and a repulsive force. (c) Output voltages. (d) Output currents. (e) Contact-separation times for C-TENG and MC-TENG.

Figure 2(a) shows schematic diagrams of a C-TENG with a mechanical contact cam and an MC-TENG with a magnetic non-contact cam. Both have the same coil springs with a stiffness of 0.5 N/mm for the same repulsive force (*F*_*r*_). The initial state of the top plate is set in such a way that the springs are compressed by 1 mm to ensure that the springs are under compression during the operation of the TENGs. The measured pushing force (*F*_*p*_) by the mechanical cam contact of the C-TENG was 3.92 N, as shown in Figure 2(b). Thus, for the MC-TENG, a magnet pair of *M*_1_ − *M*_3_ was employed, and the distance *d*_*m*1_ was set to 2 mm to create the nearly same experimental conditions for the MC-TENG and the C-TENG. When the tribo-surfaces of the MC-TENG were contacted, the distance between the magnets was 3 mm because the gap distance (*d*_gap_) between the two tribo-surfaces was 1 mm. According to the data in Figure 1(d), the measured pushing force was 4.02 N, which was almost identical with the mechanical pushing force. The contact force (*F*_*c*_) between tribo-materials can be determined by Equation (1):

(1) $$\begin{array}{c} F_{c}=F_{p}-F_{r}, \end{array}$$

Thus, all the experimental conditions were controlled to be the nearly same except for the contact mechanism (i.e., mechanical cam contact or magnetic cam contact).

Interestingly, the results showed a remarkable enhancement of the power output, as depicted in Figures 2(c) and 2(d). The averaged peak values of the voltage and current were 21.52 V and 1.02 *μ*A for the C-TENG, respectively, and 46.83 V and 2.15 *μ*A for the MC-TENG, respectively. The enhancements were approximately 217% and 211% for the voltage and current, respectively. Accordingly, the calculated values of the power output were 21.95 *μ*W and 100.68 *μ*W for the C-TENG and MC-TENG, respectively. The enhancement was approximately 458%. Since we set the nearly same contact force in the C-TENG and the MC-TENG, the enhancement should have come from the reduction of the Δ*t*. According to the previous research [30], the short circuit current *I*_sc_ is determined by

(2) $$\begin{array}{c} I_{sc}=\frac{S\sigma \left(d/\varepsilon_{r}\right)}{{\left(\left(d/\varepsilon_{r}\right)+x\left(t\right)\right)}^{2}}∙v\left(t\right), \end{array}$$

where *S* is the contact area, *σ* is the total static tribo-charge of the tribo-surfaces caused by electrostatic and contact electrification, *ε*_*r*_ is the relative dielectric constant, *d* is the thickness of the electron acceptor, and *x*(*t*) and *v*(*t*) are the distance and velocity between the acceptor and the bottom electrode, respectively. From Equation (2), we can see that the output current was dependent on the contact and separating velocities of the two tribo-surfaces. As found in Figure 2(e) of the voltage output magnified in the time span, Δ*t*_mag_ of the MC-TENG was half Δ*t*_mech_ of the C-TENG. The mechanical contact of the C-TENG was the rigid cam slipping on the rigid substrate of the top plate. Thus, the motion of the top plate was mostly dependent of the circumferential shape of the cam. If the cam was too small, the tribo-surfaces would not fully contact, and if it was too large it would stick and not be able to rotate, as shown in Figure S1(c).

### 2.3. Contact Force Effects in the MC-TENG

**Figure 3:**
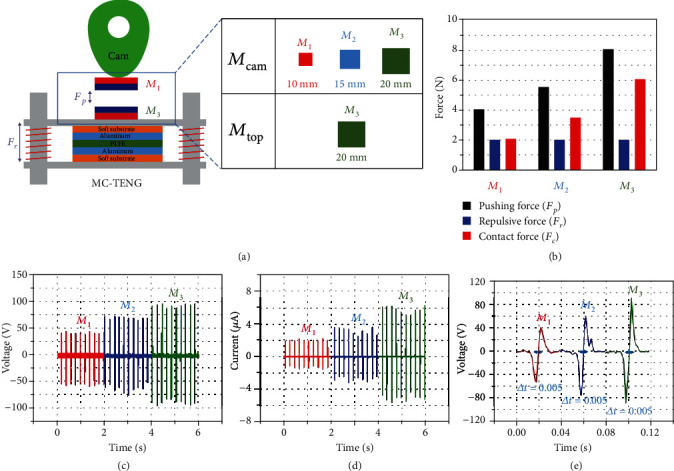
Effect of pushing force in MC-TENG. (a) Controlled pushing force by different magnets (*M*_1_, *M*_2_, and *M*_3_) on a cam. (b) Increased contact force by increasing pushing forces with different magnets. (c) Corresponding output voltages. (d) Output currents. (e) Contact-separation times in MC-TENG.

As mentioned in the Introduction, we could control the contact force in the MC-TENG without any sticking problems.  Figure 3(a) shows different combinations of the magnet pairs between *M*_cam_ and *M*_top_. When *M*_top_ was set to the *M*_3_ size, the pushing force increased as the size of *M*_cam_ changed from *M*_1_ to *M*_3_, as shown in Figure 3(b). The power output of the MC-TENG increased in accordance with the increase of the pushing force, as shown in Figures 3(c) and 3(d). The averaged peak values of the voltage output were 46.83, 64.4, and 88.0 V, respectively. The averaged peak values of the current output were 1.62, 2.84, and 5.26 *μ*A, respectively. The contact-separation time was the same value of 0.005 s for all cases because the rotating speed of the cam was kept constant, as shown in Figure 3(e).

The increased power output seems obvious because a large pushing force indicates large energy input from the cam. But in this study, we tried to demonstrate that the pushing force could be increased by controlling magnetic force, where a cam stuck problem was not occurred. In the case of the mechanical cam contact, the moment of inertia of the cam can be changed to create different pushing forces. With the same rotating frequency, the larger (i.e., longer) cam has more kinetic energy, resulting in a larger pushing force when contact the top plate. However, changing the cam size causes a stuck problem in a C-TENG. Thus, mechanical cam is limited in a certain maximum pushing force. However, changing the pushing force in the MC-TENG can be accomplished by simply replacing the permanent magnet with a different size. This change of the magnet size does not rearrange the cam and the TENG. Since there is no limitation on the fabrication of magnets in different sizes, various pushing forces can be achieved according to the change of the energy source or the external load.

### 2.4. Behaviors of Magnetic Spring

Thus far, we have used mechanical coil springs to maintain the same repulsive force; by changing the spring stiffness, we could control the repulsive force. Here follows our evaluation of magnetic springs which can replace coil springs. In mechanical coil springs, the dimensions and/or material can be changed to create different repulsive forces, which requires a reassembly process. Similarly, if the length of a coil spring is changed, a rearrangement is required, including the rotating cam; furthermore, coil springs can develop some mechanical fatigue problems. However, magnetic springs can easily control the repulsive force and can be conveniently replaced with other ones of different sizes. This does not require any rearrangement of the system, and various repulsive forces can be achieved according to the change of the energy source or the external load, ultimately providing high sustainability and low-cost maintenance.

**Figure 4:**
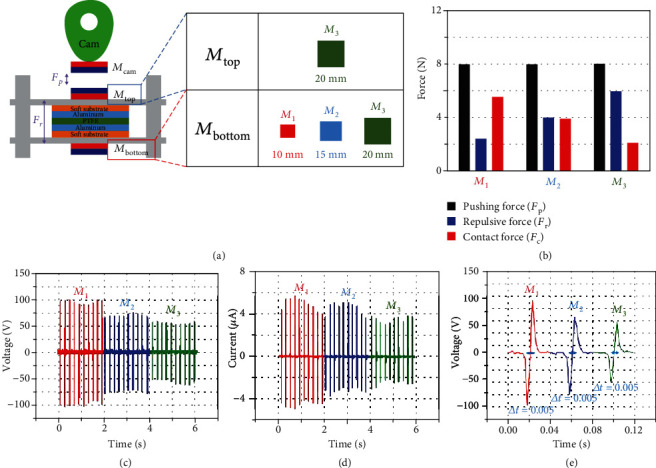
Effect of magnetic spring in MC-TENG. (a) Controlled magnetic repulsive force by different magnets (*M*_1_, *M*_2_, and *M*_3_) at the bottom substrate. (b) Decreased contact force by increasing repulsive forces with different magnets. (c) Corresponding output voltages. (d) Output currents. (e) Contact-separation times in MC-TENG with a magnetic spring.

Figure 4(a) shows schematic diagrams of an MC-TENG with a magnetic spring. *M*_cam_ and *M*_top_ were initially of size *M*_3_ and the distance between them was set to 3 mm, producing a measured pushing force of 7.95 N. Then, the size of *M*_bottom_ was increased from *M*_1_ to *M*_3_ to observe the effect of the repulsive force of the magnetic spring. The distance from the *M*_top_ was set to 7 mm and all experimental conditions were controlled to be the same except for the size of *M*_bottom_. The resultant repulsive force of each case is shown in Figure 4(b). The power output decreased significantly with respect to the increase of the repulsive force, as shown in Figures 4(c) and 4(d). The contact-separation time was the same as 0.005 s for all cases because the rotating frequency of the cam was kept constant, as shown in Figure 4(e). Therefore, it seems that the change in the power output was caused by the change in the repulsive force. A previous study reported the reduction of power output of a C-TENG by the increased repulsive force of a mechanical spring, and we observed the same phenomenon when a magnetic spring was used [30]. The averaged peak values of the voltage output were 93.8, 69.6, and 54.4 V, respectively, and those of the current output were 4.79, 3.94, and 3.04 *μ*A, respectively.

### 2.5. Optimized Design for the MC-TENG

**Figure 5:**
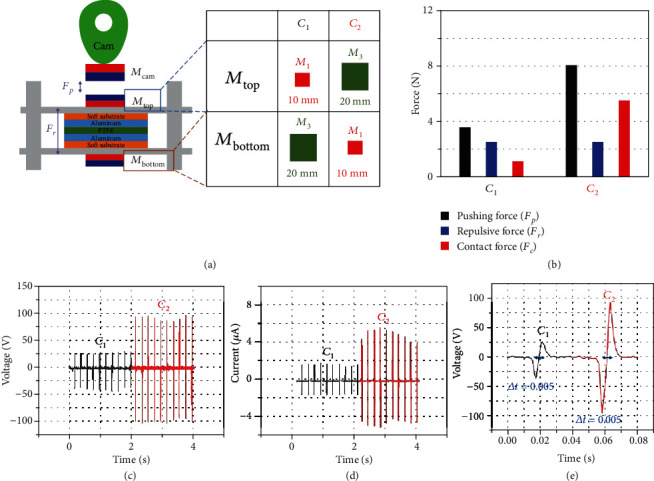
Optimized arrangement of magnets in MC-TENG. (a) Two kinds of combinations (*C*_1_ and *C*_2_) with two magnets. (b) Pushing, repulsive, and contact forces by different combinations of magnets. (c) Corresponding output voltages. (d) Output currents. (e) Contact-separation times in MC-TENG for different combinations.

We have observed that the output power of the MC-TENG could be enhanced by replacing the mechanical interaction with a magnetic interaction. In our assessments, the power output increased as the pushing force increased and the repulsive force decreased, as shown in Figures 3 and 4. In other words, the power output of the MC-TENG was much larger when the contact force was larger. To clarify the relation between the magnetic force and the power output, we performed an optimization experiment on the MC-TENG with a magnet set consisting of one *M*_3_ and one *M*_1_. As shown in Figure 5(a), two different combinations, denoted by *C*_1_ and *C*_2_, could be applied to the MC-TENG. The pushing force and repulsive force of each combination are presented in Figure 5(b), where the contact force can be determined by Equation (1).

Since the combinations *C*_1_ and *C*_2_ used the same magnets *M*_1_ and *M*_3_, the repulsive force was the same. However, the experimental results showed that combination *C*_2_ generated much more power than combination *C*_1_. The averaged peak values of voltage and current were 93.8 V and 4.79 *μ*A for combination *C*_2_, and 32.64 V and 1.61 *μ*A for combination *C*_1_, respectively. The enhancements were approximately 287% and 298% for the voltage and current, respectively. This drastic change of power output from the same magnet set was due to the different contact force. One MC-TENG can employ three permanent magnets to utilize two magnetic interaction pairs. The *M*_cam_ and *M*_top_ pair provides energy transfer by non-contact magnetic pushing force; the *M*_top_ and *M*_bottom_ pair provides the separation of the tribo-surfaces by non-contact magnetic repulsive force; and both pairs share the magnet *M*_top_, which means the design processes of the magnet pairs are coupled to each other.

The decision of which magnet pair to use for an appropriate magnetic spring affects the performance of the magnetic cam contact and vice versa. Thus, the contact force, which is defined as the difference between the pushing force of the *M*_cam_ and *M*_top_ pair and the repulsive force of the *M*_top_ and *M*_bottom_ pair, is the key factor to determine the power output of the MC-TENG. Larger contact force leads to larger power output. In accordance with the contact force, the magnets should be well arranged for the MC-TENG to generate the maximum power. The contact force in a TENG device can vary with the arrangement of the magnets, as shown in Figure S4; for example, the *C*_0_ combination shows a negative contact force value. Even though the same magnets in combinations *C*_1_ and *C*_2_ were used for the combination *C*_0_, the top plate and the bottom plate could not contact because the pushing force was too small. Finally, we could understand that the optimized arrangement of magnets should be the design with the maximum contact force (i.e., the highest pushing force and the lowest repulsive force).

### 2.6. Performance Characterizations

**Figure 6:**
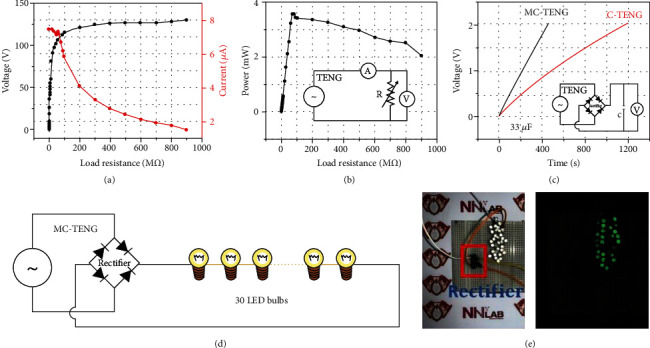
Performance demonstration of an MC-TENG application. (a) Voltage and current output under various load resistances. (b) Output power according to load resistances. (c) Capacitor charging behaviors in MC-TENG. (d) Circuit diagram to operate LED bulbs. (e) 30 LED bulbs illuminated by the MC-TENG.

We assessed the performance of the MC-TENG as an electric power source, using the magnet combination that showed the highest power output. The magnet sizes *M*_cam_, *M*_top_, and *M*_bottom_ corresponded to *M*_3_, *M*_3_, and *M*_1_, respectively. Figures 6(a) and 6(b) show the voltage (*V*) and current (*I*) measured according to the load resistance, and the corresponding power (*P*) was calculated by *P* = *VI*. As per the electric circuit diagram of Figure 6(b), a variable resistor was used for the change of load. The voltage increased but the current decreased as the load resistance increased up to 900 M*Ω*. As a result, the maximum peak power was 3.57 mW at 70 M*Ω*.

Figure 6(c) shows the process of charging a 33 *μ*F capacitor from the completely discharged state to 2.0 V with the MC-TENG and C-TENG over time. A rectifier was used to regulate the AC output of each TENG into the DC input of the capacitor. Comparing the charging of the 33 *μ*F capacitor, the C-TENG had a charging time of 1194.68 s and the MC-TENG took 461.12 s, which was 2.59 times faster. The higher voltage output of the MC-TENG led to more efficient charging, and its higher current output produced a higher power output. This again confirmed that the MC-TENG, which replaced C-TENG's mechanical interaction parts with magnetic interaction parts, exhibited a significantly higher power output.

Figures 6(d) and 6(e) explicate use of the MC-TENG as a power source for the operation of 30 light-emitting diode (LED) bulbs connected in series. The rated power of the 30 LED bulbs series was 0.6 W, and it was confirmed that instantaneous flashing was repeated. Thus, by verifying the intermittent operation of the LED bulbs with the supplied power, we verified that the MC-TENG could be utilized in a self-powered system.

### 2.7. MC-TENG-Based Windmill System Design

**Figure 7:**
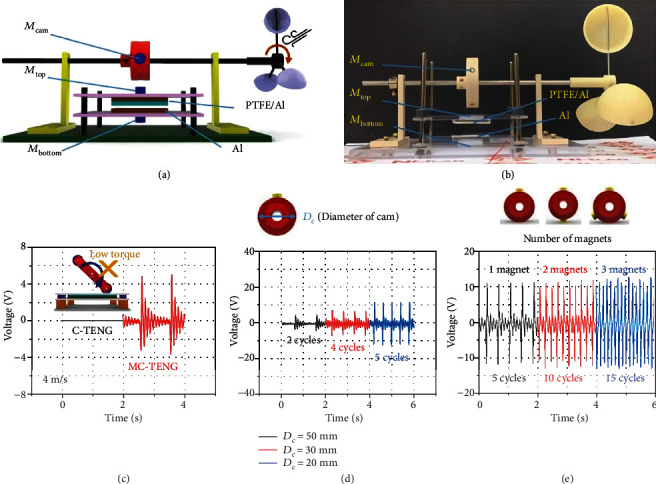
MC-TENG-based windmill system design. (a) Schematic illustration of the MC-TENG-based windmill system. (b) Photograph of MC-TENG-based windmill system. (c) Behaviors of output voltage by using C-TENG and MC-TENG under a wind speed of 4 m/s. (d) Output voltages by different cam diameters on the MC-TENG. (e) Output voltage by different numbers of magnets in the MC-TENG.

We designed and demonstrated the performance behaviors of an MC-TENG-based windmill system as an example of a practical application. When the wind blows, the windmill rotates and the connected cam also rotates. Thus, the MC-TENG could harvest energy from the wind, as shown in Figures 7(a) and 7(b). Other TENG-based windmills have been developed, but here, we focus on natural low-wind harvesting from our MC-TENG-based windmill, which can utilize a wind speed of only 4 m/s, simulating natural wind. This is the most common wind speed level in the normal environment, so the torque applied to the windmill is very low. If a C-TENG is configured in a windmill system with a 4 m/s wind speed, the windmill does not work because the cam is very likely to stick due to the low torque (Figure 7(c)). When a motor drives the cam, sufficient torque can be applied by controlling the speed of the motor, so the stuck cam problem is prevented by the precise positioning of the cam and motor. However, in a C-TENG-based windmill system, it is difficult to find the optimal operating conditions: rearrangement and reassembly are often required when changing the cam or spring. Therefore, we conducted the experiment with the same wind speed for a MC-TENG-based windmill system, and, as shown in Figure 7(c), it worked well and generated electricity.

To understand the MC-TENG-based windmill system in detail, we measured the voltage output while changing the diameter of the cam. Interestingly, when the cam diameter increased, the output performance of the MC-TENG decreased, as shown in Figure 7(d). This was caused by the diminished contact force, as simply explained by Equation (3):

(3) $$\begin{array}{c} \tau =r∙F, \end{array}$$

where *F* is the applied pushing force, *r* is the length of the torque arm (i.e., the radius of the cam), and *τ* is the resultant torque. Since the wind speed was constant, the value of the torque produced by the wind was constant even though it was low for a natural wind like 4 m/s. When the diameter of the cam increased, the torque arm increased, and accordingly, the pushing force applied to the top plate of the MC-TENG decreased. This led to decreased voltage and current output due to the decreased contact force, which is elucidated in Figures 3 and 4. This demonstrates that the output power from the MC-TENG could be additionally increased by reducing the cam size.

We also changed the number of magnets, as shown in Figure 7(e). When the number of magnets increased, the voltage output peaks occurred more frequently even though their values remained the same. More magnets made more frequent contact, but the contact velocity of the tribo-surfaces was the same because the wind speed was constant. As can be seen in Figure S5, when the number of magnets increased, the contact-separation time remained the same at 0.03 s. This again confirmed that the contact-separation time is a critical parameter to enhance output power.

## 3. Discussion

In this study, we explored the reduction of energy loss, noise, friction, shock, and damage using permanent magnets in a cam-based TENG. Even though a C-TENG is an effective design to harvest high-speed rotational energies, its poor structural sustainability might be a significant handicap in real environment applications. Therefore, we studied the performance of a magnetic force-driven, cam-based TENG (MC-TENG) considering various types of cam contact and types of repulsion springs, the contact-separation time, and the contact force. We developed a wear-free, sustainable MC-TENG by changing the mechanical interaction parts of a conventional mechanical C-TENG to magnetic interaction parts utilizing permanent magnets. We experimentally verified the relation between the contact force and the power output from the MC-TENG and found that a larger magnetic pushing force generated more electric power. The larger repulsive force of the magnetic spring produced less electric power, indicating that the lower repulsive force of the magnetic spring was better to boost the output power. The maximized electric power generation can be achieved through a suitable combination of permanent magnets that produce the maximum contact force which is the difference between the magnetic pushing force by the cam and the repulsive force by the magnetic spring. Unlike in a C-TENG, the electric power generation from an MC-TENG can be easily adjusted by replacing the permanent magnets of different sizes. It makes the design of TENG more flexible since any rearrangement or reassembly process is not required. We also demonstrated capacitor charging and LED bulb lighting performance to show the possibility of using an MC-TENG as the power source for a self-powered system. Finally, we applied an MC-TENG in a windmill system design to verify its ability to generate electric power. The benefits of its non-contact magnets and its ability to generate power at low energy input (low wind speed) proved its practical applicability. The effects of the cam diameter and the number of magnets on the power output were also demonstrated. Based on our results, we expect that our frictionless MC-TENG offers a more sustainable solution and ample output performance for effective applications in our real environment.

## 4. Materials and Methods

### 4.1. Fabrication of a Vertical Contact-Separation Type of TENG

As shown in the inset of Figure 1(a), a vertical contact-separation type of TENG is composed of top (i.e., a positive triboelectric layer) and bottom (i.e., a negative triboelectric layer) plates. The top plate is a rigid acrylic substrate under which a soft foam coated with an aluminum (Al) film at the bottom surface is attached. The soft foam has a paramount role to ensure complete contact of the tribo-surfaces, leading to the enhanced power output [30]. The bottom plate is a rigid polylactic acid (PLA) substrate on which a polytetrafluoroethylene (PTFE) layer and a soft foam coated with an Al film at the top surface in series are attached. Thus, the Al film on the top plate is the electron donor as a positive triboelectric material, and the PTFE layer on the bottom plate is the electron acceptor as a negative triboelectric material. Each tribo-surface has an area of 20 × 20 mm^2^. The bottom plate is fixed to a ground structure while the top plate has vertical motion guided by four columns posted at the vertices of the bottom plate. The gap distance between the two tribo-surfaces (*d*_gap_) was set to 1 mm. The detailed fabrication process of the TENG can be found in Figure S2.

### 4.2. Design and Fabrication of the MC-TENG

A C-TENG has two very important mechanical elements: the rotating cam for pushing and the coil springs for releasing. The kinetic energy of the rotating cam is transferred to the TENG by the mechanical contact of the cam to the top plate. The coil springs along the columns support the top plate and provide restoring force when the top plate is displaced from its initial position by the cam push (Figure S1(a)). Thus, the pushing force of the cam and the stiffness of the springs are closely related to the power output of the C-TENG. Since both the contacting cam and the springs are mechanical parts, they have problems of wear and change of characteristics over time. Especially, mechanical contact between the cam and the rigid substrate can cause noise, shock, and damage to both components, which can lead to C-TENG failure as shown in Figure S1.

As shown in Figures 1(a) and 1(b), the MC-TENG employs three permanent magnets, replacing the mechanical interactions of the C-TENG with magnetic interactions. We used rare earth neodymium-iron-boron (NdFeB) permanent magnets to obtain a large magnetic force at a small size. The magnet grade was N-30 and the sizes were 10, 15, and 20 mm (Figure 1(c)). The magnets are nickel coated to protect them from corrosion by moisture. The maximum working temperature is approximately 80°C. If the MC-TENG is kept in dry conditions and room temperature, the magnets theoretically can retain their magnetism indefinitely. The magnetic field by surrounding magnets generating repulsive force is not enough to demagnetize. The magnet attached to the tip of the cam is *M*_cam_, the magnet attached to the top surface of the top plate is *M*_top_, and the magnet attached to the bottom surface of the bottom plate is *M*_bottom_. When *M*_cam_ approaches *M*_top_ by rotation of the cam, the polarity of both magnets facing each other is the same. Then, the generated repulsive force between *M*_cam_ and *M*_top_ makes the top plate move downward and contact the bottom plate. *M*_bottom_ is placed in such a way that the same polarity is facing toward *M*_top_. When the top plate approaches the bottom plate, the generated repulsive force between *M*_top_ and *M*_bottom_ increases. Since the magnetic force between the permanent magnets exponentially increases with respect to the reciprocal of the distance [31], all magnets in an MC-TENG barely contact the other magnets. Therefore, the mechanical contacts of a C-TENG are eliminated in the MC-TENG, thus resulting in superior sustainability.

### 4.3. Characterizations and Measurements

A rotary motor (TM10-A0723, TM TECH-i) fixed to the ground was used to drive the cam, and the rotating speed was set to 300 rpm. An oscilloscope (MDO3052, Tektronix) and a low-noise current preamplifier (SR570, Stanford Research Systems) were used to measure the voltage and current output, respectively. The output values were recorded for 10 rotations of the cam by 1 kHz sampling, and they were evaluated by averaging the peak values for comparison. The contact-separation time was measured from the voltage output data by taking the time interval from a positive peak to the next negative peak, since the positive and negative voltage output is related to the contact and separation of the tribo-surfaces, respectively. A load cell (CBMS-30, Bongshin Loadcell) and a load indicator (BS-3520, Bongshin Loadcell) were used to measure the repulsive force of the permanent magnets and the mechanical force of the cam and springs. A high-speed camera (M110, Phantom) was used to observe the movement of the tribo-surfaces of the C-TENG and MC-TENG.

## Data Availability

The data used to support the findings of this study are available from the corresponding author upon reasonable request.

## Supplementary Materials

Supplementary 1 Figure S1: limitations of mechanical cam based TENG (C-TENG). Figure S2: fabrication process of C-TENG and MC-TENG. Figure S3: experimental setup for magnetic force measurement. Figure S4: understanding for optimized arrangement of magnets in MC-TENG. Figure S5: contact-separation times (Δ*t*) for different numbers of magnets in an MC-TENG-based windmill system, where Δ*t* was all the same.
