# Supplementary material for: Magnetic Force Enhanced Sustainability and Power of Cam-Based Triboelectric Nanogenerator
Source: Research (Wash D C). 2021 Mar 8;2021:6426130. doi: 10.34133/2021/6426130 (PMC7968483; doi:10.34133/2021/6426130)
Supplement: Supplementary 1 — Figure S1: limitations of mechanical cam based TENG (C-TENG). Figure S2: fabrication process of C-TENG and MC-TENG. Figure S3: experimental setup for magnetic force measurement. Figure S4: understanding for optimized arrangement of magnets in MC-TENG. Figure S5: contact-separation times (Δt) for different numbers of magnets in an MC-TENG-based windmill system, where Δt was all the same. [file 6426130.f1.zip › Revised Supplementary Materials (with Highlighted Changes).docx]

**Supplementary Information**

**Magnetic Force enhanced Sustainability and Power of Cam based Triboelectric Nanogenerator**

Hakjeong Kim^1^, Hee Jae Hwang^1^, Nghia Dinh Huynh^1^, Khanh Duy Pham^1^, Kyungwho Choi^2^, Dahoon Ahn^3*^, Dukhyun Choi^1*^

^1^Department of Mechanical Engineering (Integrated Engineering Program), Kyung Hee University, Yongin 17104, Republic of Korea

^2^Department of Mechanical Engineering, Korea Aerospace University, Goyang 10540, Republic of Korea

^3^Division of Mechanical Engineering, Kongju National University, Cheonan 31080, Republic of Korea

Correspondence should be addressed to Dahoon Ahn; [dhahn@kongju.ac.kr](mailto:dhahn@kongju.ac.kr) and Dukhyun Choi; ([dchoi@khu.ac.kr](mailto:dchoi@khu.ac.kr))


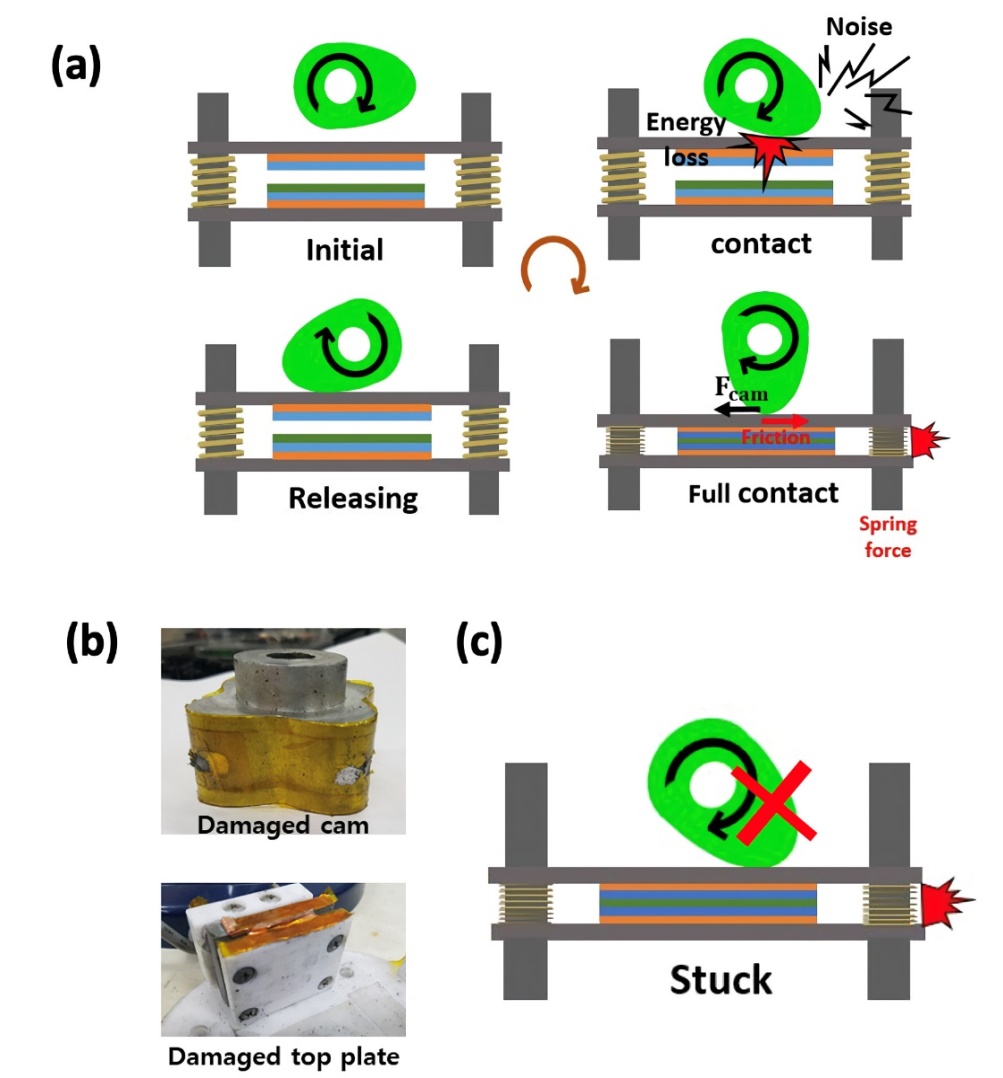


FIGURE S1. Limitations of mechanical cam based TENG (C-TENG). (a) Energy losses such as friction and noise in C-TENG. (b) Damaged cam and top plate by friction and wear when a mechanical cam was used. (c) Stuck cam problem of C-TENG with a larger cam, where a pushing force can be increased, but the cam cannot be rotated due to the stuck of a cam in C-TENG.


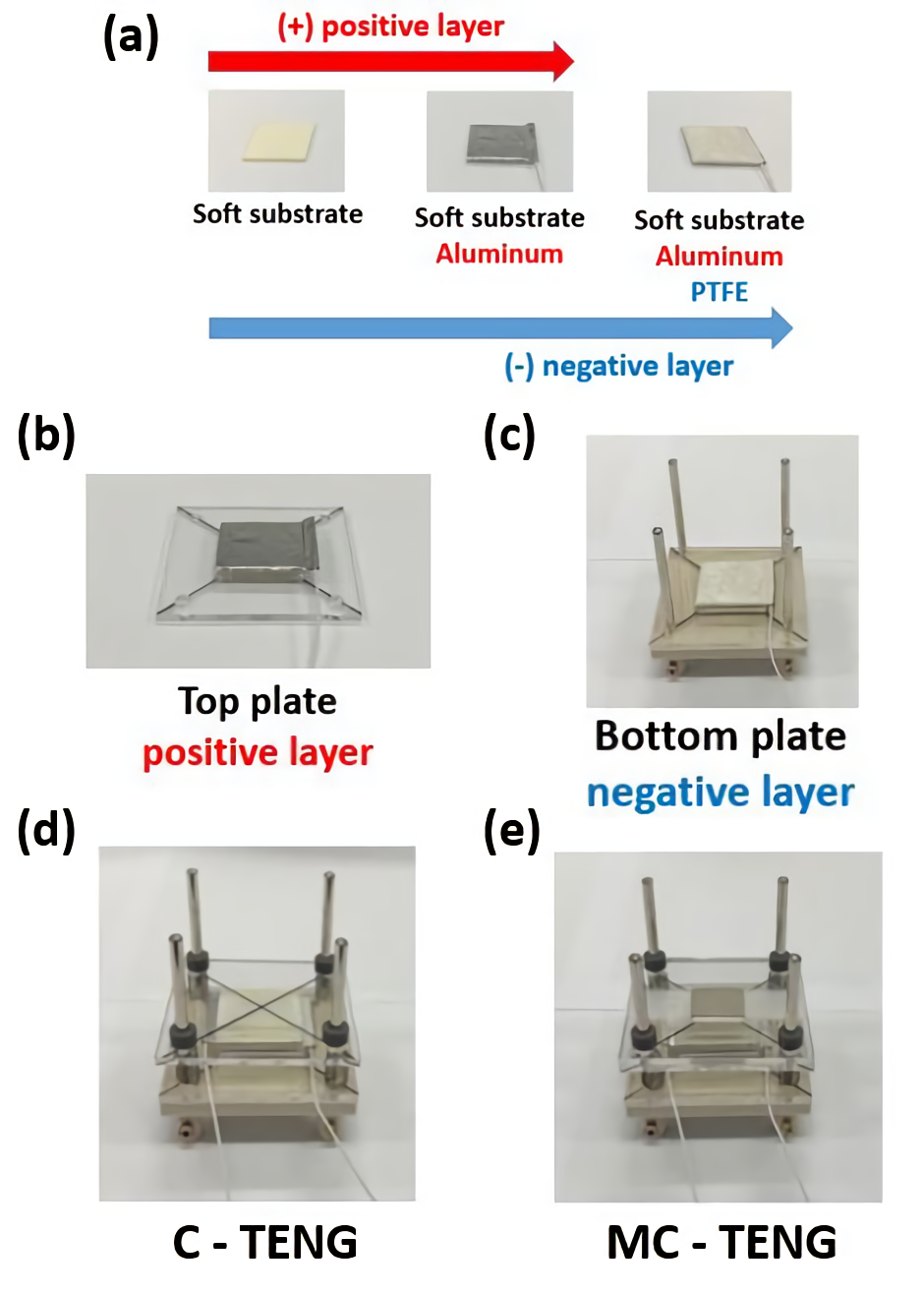


FIGURE S2. Fabrication process of C-TENG and MC-TENG. (a) Preparation of positive and negative layers on a soft substrate. (b) Top plate assembled with a positive layer. (c) Bottom plate assembled with a negative layer. Integrated photographs for (d) C-TENG and (e) MC-TENG.


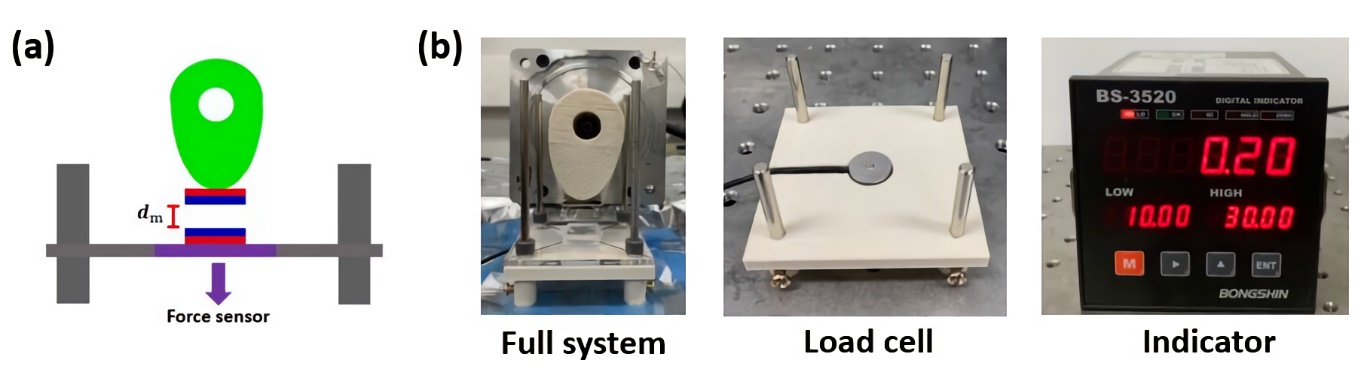


FIGURE S3. Experimental setup for magnetic force measurement. (a) Schematic illustration for the measurement of magnetic repulsive force by using a force sensor. (b) Photographs for measurement systems (a load cell and an indicator).


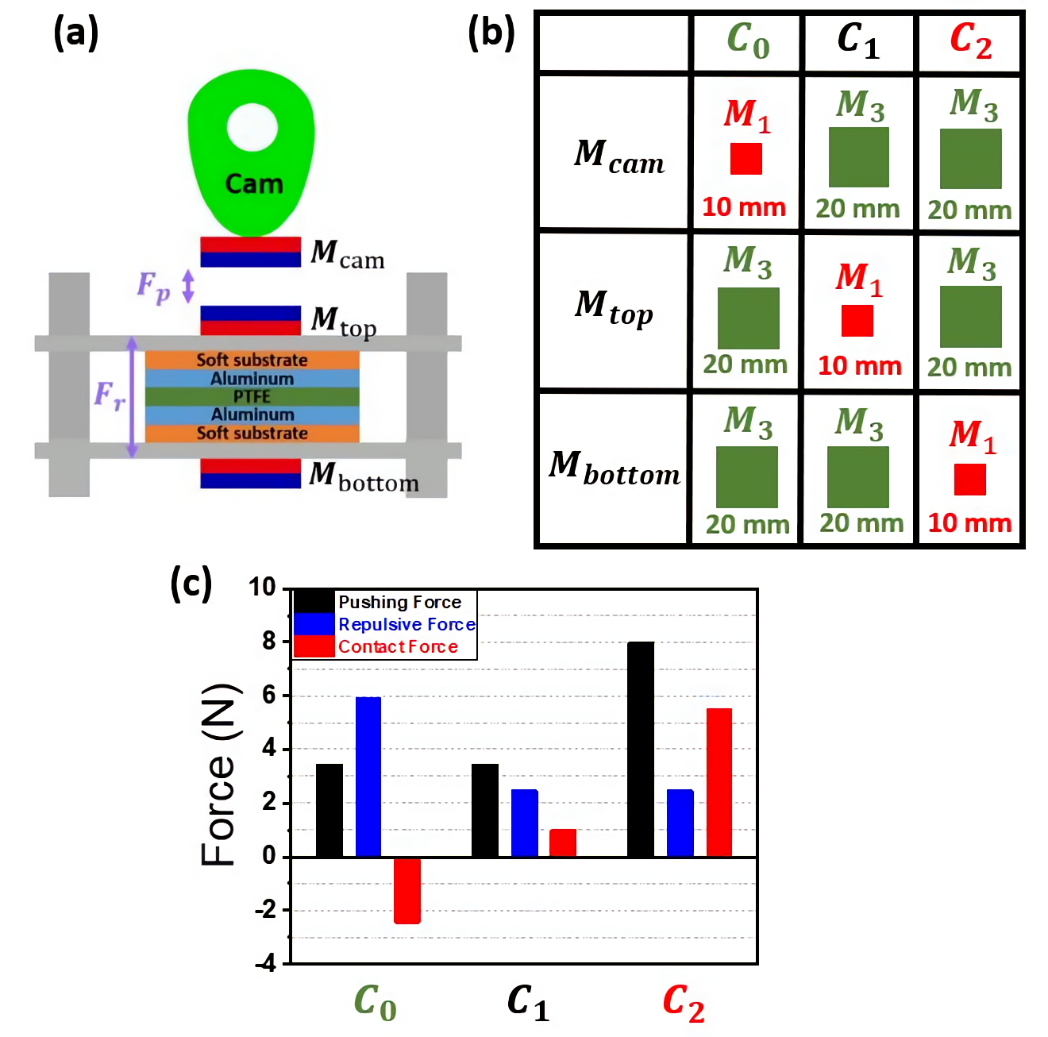


FIGURE S4. Understanding for optimized arrangement of magnets in MC-TENG. (a) Illustration of MC-TENG. (b) Three kinds of combinations (*C*_0_, *C*_1_, and *C*_2_) with two magnets (*M*_1_ and *M*_3_). (c) Pushing, repulsive, and contact forces by different combinations of magnets.


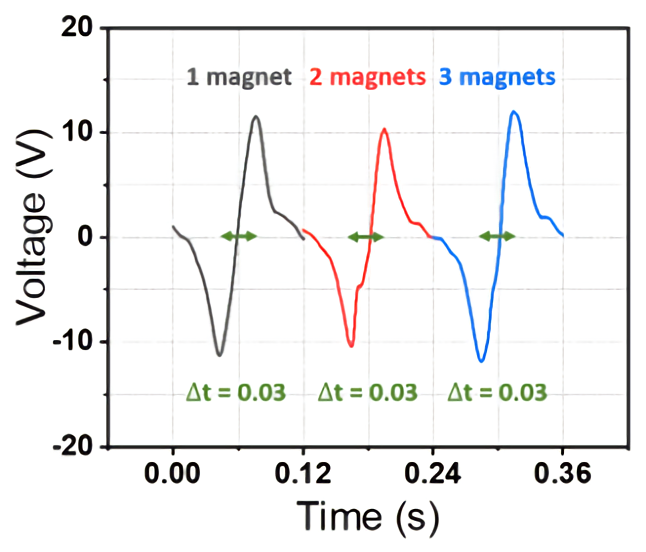


FIGURE S5. Contact-separation times (*Δt*) for different numbers of magnets in an MC-TENG-based windmill system, where *Δt* was all the same.
